# Post-COVID-19 neuropsychiatric manifestations: a suggested therapeutic approach to ‘long COVID’ with azithromycin

**DOI:** 10.1017/S0950268823001966

**Published:** 2023-12-15

**Authors:** Robert A. Schwartz, Robert M. Suskind

**Affiliations:** 1 Rutgers New Jersey Medical School, Newark, NJ, USA; 2 California University of Science and Medicine, Colton, CA, USA

**Keywords:** azithromycin, COVID-19, encephalitis, Kapila syndrome, macrolide, SARS-CoV-2, von Economo disease

## Abstract

The devastating effects of the coronavirus disease (COVID-19) caused by severe acute respiratory syndrome coronavirus 2 (SARS-CoV-2) may not end when the acute illness has terminated. A subset of COVID-19 patients may have symptoms that persist for months. This condition has been described as ‘long COVID’. From a historical perspective, it has been recognized that serious long-term neurological sequelae have been associated with RNA viruses such as influenza viruses and coronaviruses. A potential intervention for early post-COVID-19 neuropsychiatric impairment may be the commonly employed, readily available, reasonably priced macrolide antibiotic, azithromycin. We have observed a favourable clinical response with azithromycin in three patients with neurological symptoms associated with long COVID-19. We recommend considering formal clinical trials using azithromycin for patients with post-COVID-19 infection neurological changes including ‘COVID fog’ or the more severe neurological symptoms that may later develop.

## Introduction

The SARS 2 virus has caused a pandemic that has killed millions, which is comparable to the Spanish flu pandemic of 1918–1919 [1, 2]. Autopsies performed and documented by Economo on Spanish flu pandemic patients and by Kapila et al. [3] on the 1957 Asian flu pandemic patients showed distinct neuropathological changes, as did recent studies on COVID-19 patients with neuropsychiatric symptoms [1, 4–6]. While the long-term effects of COVID-19 remain unclear, they have been documented and remain of concern. With the two serious previous coronavirus infections, namely, SARS-CoV-1 and Middle East respiratory syndrome coronavirus, and the two momentous influenza pandemics of the twentieth century that killed more than a million people, findings of narcolepsy, seizures, encephalitis, encephalopathy, and other neuropsychiatric sequelae have been described [7–9]. We applaud clinical trials examining whether empiric COVID-19 therapies are associated with differences in the prevalence and outcome of severe neurological complications of COVID-19 [4, 9], and the new NIH initiative proclaimed by NIH Director Francis S. Collins to study long COVID, noting the $1.15 billion Congressional appropriation for it [10].

## Clinical experience

Numerous clinical trials on acute COVID-19 are in progress, with azithromycin alone being studied [11, 12]. We have advocated for full clinical trials to determine whether a 5-day course of azithromycin at the first sign of COVID-19 would be beneficial [11]. Based on our favourable experience with three ‘long COVID’ patients, we now suggest full clinical trials at the onset of neurological symptoms post-acute COVID-19, assuming there are no contraindications to the use of azithromycin such as a history of liver or heart disease or a hypersensitivity to azithromycin, erythromycin, or other macrolides.

Azithromycin, a commonly employed macrolide antibiotic, is a documented immunomodulatory agent [11]. It is considered safe, with occasional urticaria and other cutaneous eruptions and rarely cardiac arrhythmias in the elderly. Clinical trials with azithromycin for early COVID-19, often in combination with hydroxychloroquine, are still in progress with 25 listed for the US alone on the ClinicalTrials.gov US governmental website, with 8 delineated as suspended, terminated, or withdrawn [12]. An especially interesting one was the ‘Azithromycin for COVID-19 Treatment in Outpatients Nationwide (ACTION)’ based at the University of California San Francisco (ClinicalTrials.gov Identifier: NCT04332107), in collaboration with the Bill & Melinda Gates Foundation, Pfizer, and Stanford University. It is officially titled ‘Azithromycin for Prevention of Disease Progression in Patients with Mild or Moderate COVID-19’. The outcome of the innovative University of California San Francisco ACTION clinical trial using a single 1.2 g course of azithromycin could have proven significant but was terminated early for ‘futility’ on 16 March 2021.

As this novel SARS 2 coronavirus sweeps across the globe, its long-term effects are being delineated [1, 2]. Pulmonary, cardiac, cerebral, hepatic, and other end-organ complications should be carefully documented, evaluated, and monitored. To determine distinct pathological changes in end organs related to the present COVID-19 pandemic, detailed investigations should be performed on tissues at the cellular level obtained at premortem or at autopsies. Ideally, these efforts will be collaborative and worldwide. Autopsies performed and documented by Economo on the 1918 Spanish flu pandemic patients and by Kapila et al. [1–3] on the 1957 Asian flu pandemic patients showed distinct pathological changes that revealed two new syndromes, which may provide a clearer understanding of the present COVID-19 pandemic and future ones. In recent coronavirus outbreaks, neuropsychiatric symptoms tended to be overlooked in favour of respiratory and other symptoms. During the more recent influenza epidemics and other coronavirus infections (SARS-CoV-1 epidemic and the Middle East respiratory syndrome coronavirus), neuropsychiatric sequelae have been delineated, including narcolepsy, seizures, encephalitis, encephalopathy, Parkinson’s disease, and limbic system involvement producing ‘moral imbecility’ [1, 6–8].

## Conclusion

Neuropsychiatric and other sequelae of COVID-19 infection represent a major concern [10]. A number of therapeutic agents should be explored globally against the consequences of COVID-19 infection. There are encouraging anecdotal results of favourable clinical experiences using azithromycin alone not only early in the course of a COVID-19 infection but in its aftermath. Accordingly, we suggest clinical trials to assess the use of azithromycin alone for long COVID-19.

As previously noted [11], we suggest formal clinical trials utilizing the prepackaged formulation of azithromycin given as soon as COVID-19 infection appears, starting with a five-day intervention of 500 mg the first day and 250 mg for the remaining four days, with a total of 1.5 g for adults older than 18 years, and a paediatric dosing of 10 mg/kg on the first day and 5 mg/kg for the remaining four days for children aged 5 to 18 years. We suggest the same regimen be considered at the first sign of long COVID. Documented laboratory diagnosis of COVID-19 infections is necessary, with patients evaluated for coinfections with other respiratory pathogens, including seasonal influenza, multi-drug-resistant *Candida auris*, and azithromycin-sensitive *Mycoplasma pneumoniae* [13].

Long COVID or post-acute sequelae of COVID-19 require our scrutiny. Clearly, it has the potential neurological and psychiatric features long established with RNA viruses such as influenza virus and, more recently, with coronaviruses [14, 15]. Since scientific evidence is weak at this time [16–20], we strongly encourage clinical trials on long COVID with azithromycin to confirm or refute the value of this potentially efficient, cost-effective, readily available, easy-to-use, and relatively safe medication.

## Data Availability

The supporting data are available on request from the corresponding author.
